# An innovative model for conductivity of graphene-based system by networked nano-sheets, interphase and tunneling zone

**DOI:** 10.1038/s41598-022-19479-9

**Published:** 2022-09-07

**Authors:** Yasser Zare, Kyong Yop Rhee

**Affiliations:** 1 Biomaterials and Tissue Engineering Research Group, Department of Interdisciplinary Technologies, Breast Cancer Research Center, Motamed Cancer Institute, ACECR, Tehran, Iran; 2 Department of Mechanical Engineering (BK21 Four), College of Engineering, Kyung Hee University, Yongin, Republic of Korea

**Keywords:** Materials science, Physics

## Abstract

This study presents a simple equation for the conductivity of graphene-filled nanocomposites by considering graphene size, amount of filler in the net, interphase deepness, tunneling size, and properties of the net. The amount of nanoparticles in the net is related to the percolation threshold and effective filler content. The novel model is analyzed using the measured conductivity of numerous examples and the factors’ impacts on the conductivity. Both experienced data and parametric examinations verify the correctness of the novel model. Among the studied factors, filler amount and interphase deepness implicitly manage the conductivity from 0 to 7 S/m. It is explained that the interphase amount affects the operative quantity of nanofiller, percolation threshold, and amount of nets.

## Introduction

Graphene nano-sheets in one or several flat layers of carbon particles tightly crowded in a two-dimensional (2D) space effectively increase the electrical conductivity of matrices^1–9^. The high aspect ratio (ratio of diameter to thickness) of graphene causes an extremely low percolation threshold, which can cause significant conductivity by low filler concentration. Hence, graphene-based nanocomposites are perfect for fabricating low-cost and efficient sensors, primarily owing to their unique properties, such as high sensitivity, good selectivity, and low-limit detection^10–15^.

Obtaining high-quality graphene in substantial quantities and the uniform dispersion of graphene in the matrices are major challenges to realize its excellent properties^7,16–21^. Previous studies have tried to overcome these problems by producing nanocomposites exhibiting high conductivity by low filler concentration. The nanocomposite containing graphene suggests a lower percolation threshold and better conductivity compared to CNT samples^22^, primarily owing to the large aspect ratio and immense specific superficial area of graphene nano-sheets^23,24^, although aggregation/agglomeration, crimping, and hard nets of graphene may diminish its efficiency^25^.

Many parameters control the conductivity of systems, such as the amount, conduction, size, and dispersion of nanofiller beside the interphase section^26–32^. There are many models that can predict the conductivity of CNT polymer products^33,34^. The models assume the effects of a tunneling mechanism, polymer-filler interfacial energy, accumulation, and curliness of CNT on the conductivity^35–38^. However, there are limited models for the conductivity of polymer graphene nanocomposite. The conductivity of polymer graphene nanocomposite was extensively evaluated using a simple power-law equation^39–41^; however, the power equation cannot consider the innovative and attractive facets of nanoparticles in conductivity.

The interphase nearby the nanofiller primarily affects the properties of systems^42,43^. The impact of interphase on the rigidity of nanocomposites has been reported^44–46^. Moreover, the interphase can provide the continuous nets in the system, which speeds up the percolation threshold, thereby growing the nets^47,48^. However, it warrants further study. In addition, electron tunneling primarily manages the electrical conductivity of polymer nanocomposite, since the electrons can be transported among adjoining nanoparticles through the tunneling effect^49–52^. Consequently, the nanoparticles can cause conductivity even when they are separated by a small distance. Nevertheless, the existing models for the conductivity of graphene systems have not considered the roles of these key parameters.

In this study, a model is proposed for the conductivity of CNT systems by considering the terms for the nanocomposites containing graphene. In fact, an innovative model is developed by considering net parameters, filler conduction, the volume portion of nanoparticles belonging to the conductive nets, and tunneling distance. Furthermore, the percolation threshold and effective quantity of graphene are correlated to the filler size, interphase deepness, and tunneling size. Additionally, the net parameter is defined expressing the dimensionality and density of the nets in the product. Therefore, the developed model associates the conductivity of nanocomposite to net properties, net percentage, effective filler concentration, interphase depth, graphene conduction, tunneling distance, and graphene dimensions. The innovative model is analyzed using the tested data of several samples from previous studies and analysis of factors.

## Equations

A linear equation for conductivity of CNT-based systems (erratically distributed CNT) was offered^53^ as:

1 $$\sigma = \sigma_{0} + \frac{{f\phi_{f} \sigma_{f} }}{3}$$

where “σ_0_” and “σ_f_” denote the conductivity of polymer medium and nanofiller, respectively, “f” denotes the percentage of nanosheets in the network after percolation threshold and “$\phi_{f}$” indicates the filler volume share. “σ_0_” is approximately 10^−15^ S/m, which is marginalized. Moreover, it was observed in previous studies that the conductivity indicates a rapid increment at the percolation threshold^54–56^; so, it cannot linearly depend on the filler concentration. Besides, this equation improperly over-estimates the conductivity of graphene-filled samples, primarily because it neglects the effects of some critical issues, such as interphase, tunnels, and net.

The interphase regions around nanoparticles can add to the filler nets, and they raise the filler efficiency of the product. The volume percentage of interphase in graphene systems^57^ is calculated as follows:

2 $$\phi_{i} = \phi_{f} \left( {\frac{{2t_{i} }}{t}} \right)$$

where “t” and “t_i_” denote the thickness of graphene nano-sheets and interphase, respectively.

The operative amount of graphene in the samples is provided by the following:

3 $$\phi_{eff} = \phi_{f} + \phi_{i} = \phi_{f} \left( {1 + \frac{{2t_{i} }}{t}} \right)$$

Moreover, the electrical conductivity in graphene nanocomposite is controlled by tunneling where electrons are moved via neighboring sheets^58–61^. Consequently, the tunneling distance (d) affects the conductivity of samples.

The percolation threshold in graphite samples (randomly arranged particles) was suggested^62^ as:

4 $$\phi_{p} = \frac{{27\pi D^{2} t}}{{4(D + d)^{3} }}$$

where “D” denotes the diameter of nano-sheets. D ≫ d eliminates the impact of tunneling size, which results in:

5 $$\phi_{p} = \frac{27\pi t}{{4D}}$$

As mentioned previously, the interphase and tunnels desirably change the percolation threshold. Therefore, the latter equation can be developed assuming the roles of these parameters as:

6 $$\phi_{pi} = \frac{27\pi t}{{4D + 2(Dt_{i} + Dd)}}$$

In addition to linking to the geometry of nano-sheets, the percolation threshold also correlates to the interphase deepness and tunneling size.

Furthermore, only the graphene nano-sheets in the nets can affect the conductivity by moving electrons.

The ratio of CNT participating in the nets^63^ can be written as:

7 $$f = \frac{{\phi_{f}^{1/3} - \phi_{p}^{1/3} }}{{1 - \phi_{p}^{1/3} }}$$

which can be applied to forecast the percentage of graphene sheets in the nets.

The interphase and tunnels handle the effective filler amount and percolation threshold modifying the “f” as:

8 $$f = \frac{{\phi_{eff}^{1/3} - \phi_{pi}^{1/3} }}{{1 - \phi_{pi}^{1/3} }}$$

The volume fraction of nano-sheets precipitating in the conductive nets is calculated by “f” as:

9 $$\phi_{N}^{{}} = f\phi_{eff}^{{}}$$

**Figure 1:**
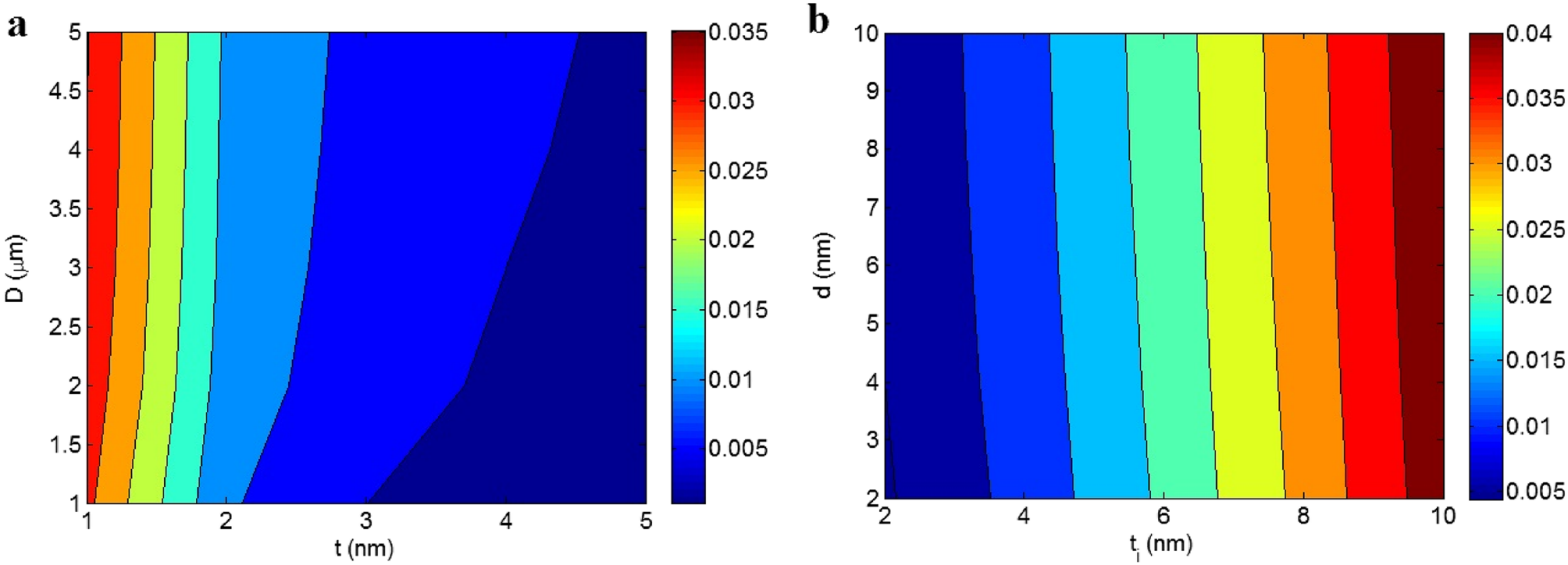
Contour plots for the dependencies of “$\phi_{N}$” on (**a**) “t” and “D” and (**b**) “t_i_” and “d”.

Figure 1a illustrates the impacts of “t” and “D” on “$\phi_{N}$” at $\phi_{f}$ = 0.01, t_i_ = 4 nm, and d = 5 nm by contour plot. As observed, the thin and large nano-sheets produce the highest “$\phi_{N}$” as 0.035, while thick and short ones decrease the “$\phi_{N}$” to almost zero. Moreover, the impacts of “t_i_” and “d” on “$\phi_{N}$” at $\phi_{f}$ = 0.01, t = 2 nm and D = 2 μm are presented in Fig. 1b. “$\phi_{N}$” primarily depends on “t_i_”, and “d” has a negligibly effect. As shown, $\phi_{N}$ = 0.04 is observed at t_i_ > 9.5 nm, while $\phi_{N}$ = 0.005 is produced by t_i_ = d = 2 nm. Therefore, thin and large nano-sheets plus a deep interphase can effectively govern the amount of networked graphene.

Furthermore, the size, dimensionality, and compactness of nets affect the conductivity. However, existing models have mostly disregarded their roles. Although it is difficult to characterize the properties of nets, it is known that these parameters directly affect the conductivity. Accordingly, a definite and dimensionless factor (K) can be suggested as a net parameter to consider the impacts of net properties on the conductivity. The higher the “K”, the larger and denser the network causing higher conductivity in nanocomposites.

Based on the above clarifications, Eq. (1) can be developed as:

10 $$\sigma = \frac{{K(f\phi_{eff}^{{}} )^{2} \sigma_{f} }}{{3\left( \frac{d}{z} \right)^{3} }}$$

where “z” denotes a tunneling parameter as 1 nm. Equation (10) indicates the effects of the net properties, percentage of nanoparticles in the nets, effective filler concentration, interphase thickness, graphene conduction, tunneling distance, and graphene dimensions on the conductivity.

The innovative model can be expressed using the volume fraction of networked graphene (Eq. 9), as:

11 $$\sigma = \frac{{K\phi_{N}^{2} \sigma_{f} }}{{3\left( \frac{d}{z} \right)^{3} }}$$

which is an accurate expression for the samples containing randomly arranged nano-sheets in the polymer matrix. The developed equations are examined in the next section using experimental data and theoretical calculations.

## Results and discussion

First, the innovative equations and model are evaluated using the tested results for the samples from former articles, because considerable experimental data for various types of graphene-filled samples are required to confirm the developed model. The details on how the data were acquired, how graphene was mixed with the polymers, and the characterization of graphene dimensions were reported in the references.

**Figure 2:**
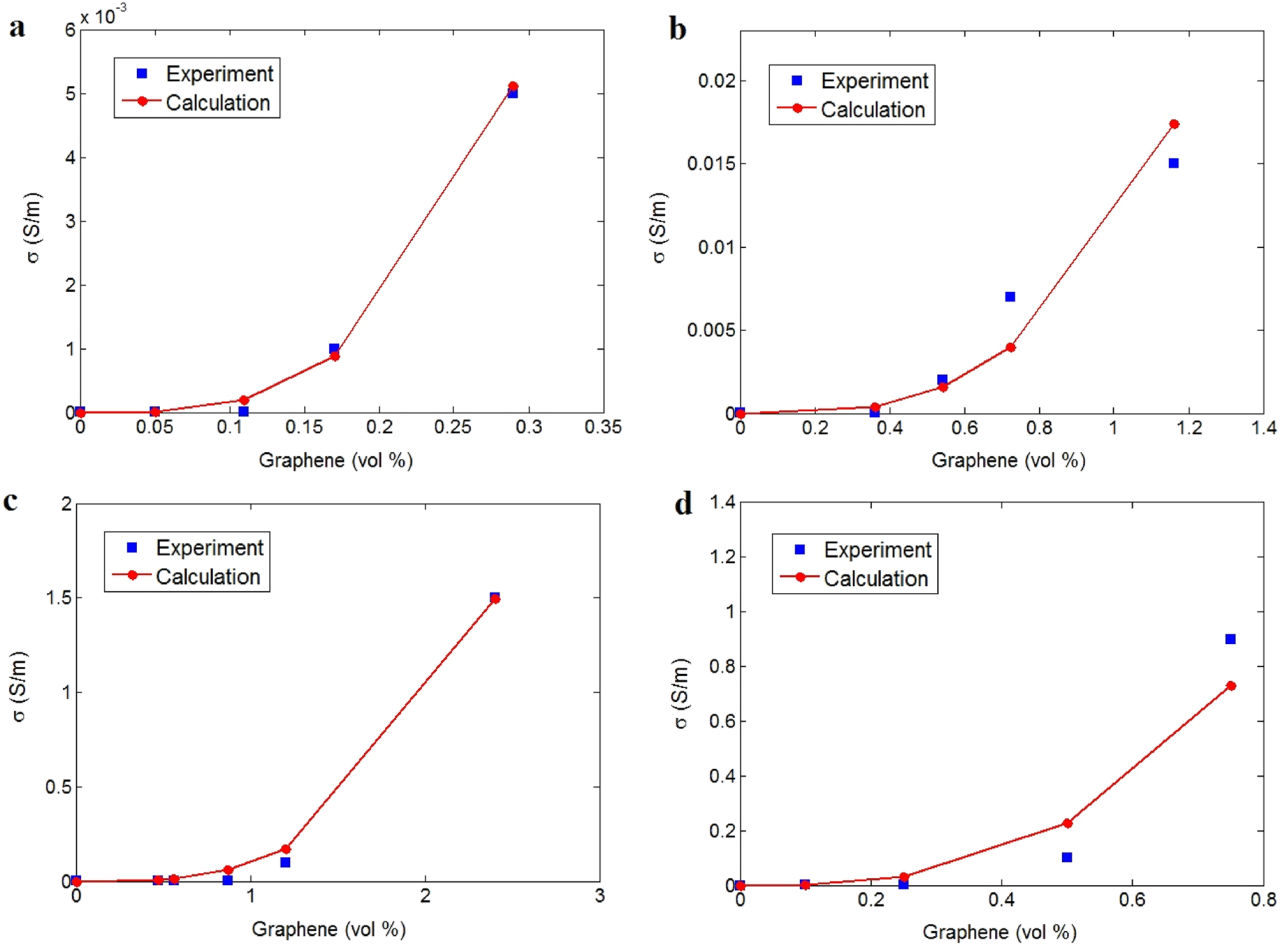
Tested conductivity and forecasts for (**a**) PI^54^, (**b**) PVA^55^, (**c**) PET^64^, and (**d**) PS^56^ graphene products.

Figure 2 presents the average measurements and forecasts of conductivity for different graphene samples: PI (D = 5 μm and t = 3 nm)^54^, PVA (D = 2 μm and t = 2 nm)^55^, PET (D = 2 μm and t = 2 nm)^64^, and PS (D = 2 μm and t = 1 nm)^56^. It can be observed that the innovative model expresses satisfactory predictions compared to tested data. Consequently, the experimental results confirm the precision of the novel model for polymer graphene materials. The extents of interphase deepness, tunneling distance, and “K” net parameter are determined using advanced equations.

Here, “t_i_” and “d” were calculated by fitting the measured percolation threshold of samples to Eq. (6). The percolation threshold was determined as 0.0015, 0.0035, 0.005, and 0.001 for PI, PVA, PET, and PS nanocomposites, respectively. Applying these levels to Eq. (6) results in the calculations of (t_i_, d) levels as (7, 9), (5, 5), (3, 4), and (8, 8) nm for the reported samples, respectively. Accordingly, PI and PS nanocomposites exhibit the densest interphase and the biggest tunnels, since they exhibit the smallest percolation threshold among the specimens.

Furthermore, the values of “K” representing the properties of conductive nets can be calculated when the measurements of conductivity are used in the novel model. The “K” values are obtained as 16, 0.14, 2.6, and 3.5 for PI/graphene, PVA/graphene, PET/graphene, and PS/graphene samples, respectively. Therefore, it can be concluded that the most efficient nets are shown in the PI/graphene sample, while PVA/graphene nanocomposite displays the weakest ones. The results for “K” as the properties of nets conform to the percolation threshold and size of interphase and tunnels. The best nets are observed in PI/graphene sample based on “K” level. Moreover, thick interphase and large tunneling distance produce the most efficient nets calculated for the considered sample. Accordingly, the percolation threshold, interphase deepness, tunneling size, and “K” net parameter exhibit similar trends, which approve the correctness of the proposed equations in this study. Decisively, the proposed equations can forecast the mentioned parameters in the graphene system by the experimented conductivity.

In the second step, the impacts of all variables on the conductivity are investigated to indorse the predictability of the novel model.

**Figure 3:**
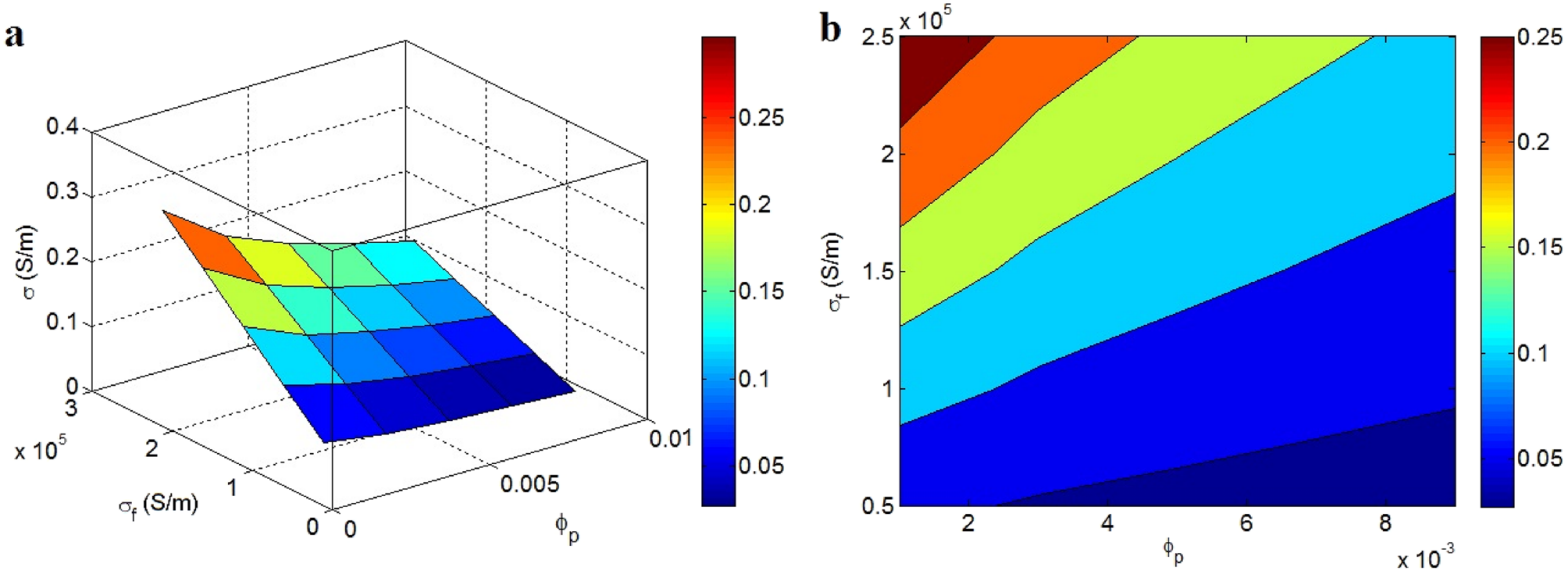
Conductivity of nanocomposite at altered levels of “$\phi_{p}$” and “σ_f_” ($\phi_{f}$ = 0.01, t = 2 nm, t_i_ = 4 nm, d = 5 nm and K = 2) by (**a**) 3D and (**b**) contour schemes.

Figure 3 presents the impacts of “$\phi_{p}$” and “σ_f_” on the conductivity at t = 2 nm, $\phi_{f}$ = 0.01, t_i_ = 4 nm, d = 5 nm, and K = 2. The conductivity of 0.25 S/m is acquired at $\phi_{p}$ = 0.001 and σ_f_ = 2.5 × 10^5^ S/m, while $\phi_{p}$ > 0.006 and σ_f_ < 0.7 × 10^5^ S/m yield the conductivity of 0.02 S/m. Accordingly, the least percolation threshold and the highest graphene conduction cause the best conductivity. In contrast, a low conductivity can be detected at a high percolation threshold and low filler conduction.

According to Eq. (8), the percolation threshold affects the percentage of nanoparticles in the nets. In fact, a very low percolation threshold produces a high “f”, indicating the establishment of large nets in the system. Accordingly, a low percolation threshold positively affects the conductivity through the construction of big nets. Moreover, the inverse relation between nanocomposite’s conductivity and percolation threshold has also been reported using experimented and foreseen data^54–56^. Meanwhile, the high conduction of graphene obviously enhances the conductivity, primarily because the nanocomposite contains the insulated matrix and conductive graphene. In other words, only conductive nano-sheets of graphene improve the conductivity. Consequently, the innovative model correctly illustrates the inspirations of “$\phi_{p}$” and “σ_f_” on the conductivity of graphene systems.

**Figure 4:**
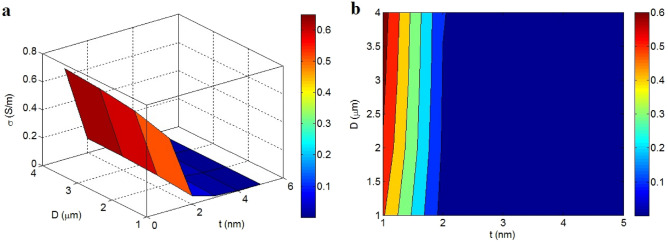
Dependencies of conductivity on the graphene size by (**a**) 3D and (**b**) contour images.

The influence of graphene size on the conductivity of the nanocomposite are depicted in Fig. 4 at constant $\phi_{f}$ = 0.01, t_i_ = 4 nm, d = 5 nm, σ_f_ = 10^5^ S/m, and K = 2. Thick nano-sheets significantly decrease the conductivity, whereas thin and large nano-sheets create high conductivity. As illustrated, t > 2 nm produce a conductivity of zero; however, t = 1 nm and D > 2.5 μm lead to a conductivity of 0.6 S/m. Resultantly, it can be stated that thin and large nano-sheets can produce a desirable conductivity in the graphene nanocomposite.

The thin and large nano-sheets produce extraordinary levels of aspect ratio and surface area, which enhance their effectiveness in conductivity. More specifically, the extensive superficial zone and large aspect ratio of nano-sheets decline the percolation threshold^65^ producing big nets, which desirably govern the conductivity. In contrast, thick nano-sheets decrease the effective volume fraction of nanoparticles (Eq. 3) by weakening the interphase volume fraction and increase the percolation threshold (Eq. 6), which negatively impact the conductivity. Therefore, the novel model correctly expresses the impacts of filler dimensions on the conductivity.

**Figure 5:**
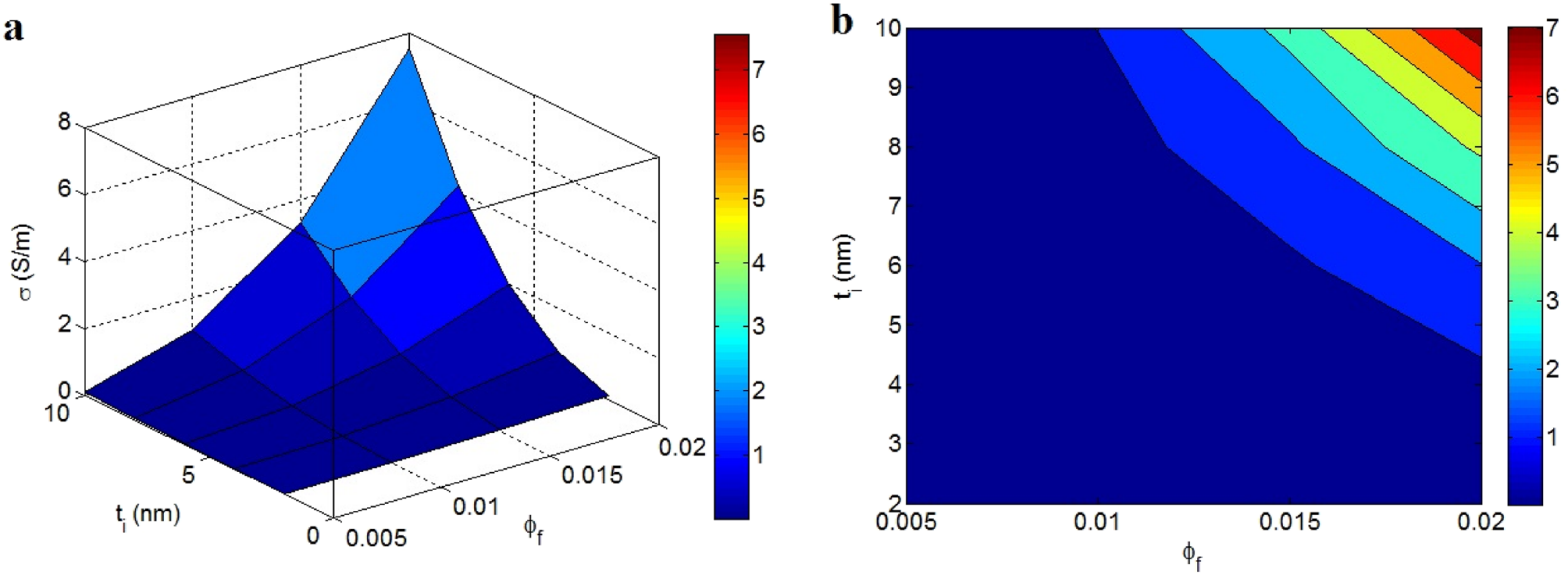
Impacts of “$\phi_{f}$” and “t_i_” on the conductivity in t = 2 nm, D = 2 μm, σ_f_ = 10^5^ S/m, d = 5 nm and K = 2 by (**a**) 3D and (**b**) contour schemes.

Figure 5 illustrates the powers of “$\phi_{f}$” and “t_i_” on the conductivity at t = 2 nm, D = 2 μm, d = 5 nm, σ_f_ = 10^5^ S/m, and K = 2. The large heights of these factors significantly augment the conductivity, nevertheless the small and medium values of both “$\phi_{f}$” and “t_i_” result in poor conductivity. Hence, both “$\phi_{f}$” and “t_i_” as filler volume fraction and interphase deepness affect the conductivity, where a supreme conductivity of 7 S/m was obtained at $\phi_{f}$ = 0.02 and t_i_ = 10 nm.

Indeed, the concentration of conductive phase (graphene nanoparticles) recovers the conductivity of the entire product, primarily because the polymer is insulated. Therefore, it is logical to observe a high conductivity at high filler concentrations. In contrast, a profuse interphase near the filler positively changes the percolation threshold (Eq. 6) and enhances the scale of conductive nets (Eq. 8), because the interphase regions are involved in the net structures. Accordingly, a thick interphase can provide positive conditions for the conductivity of nanocomposite by enhancing the networked nanoparticles. Conversely, skinny interphase cannot change the percolation threshold and the amount of nets; consequently it has a negligible impact on the conductivity. Thus, the innovative model suitably expresses the dependency of conductivity on the interphase thickness. Reportedly, the interphase roles in the thermal and electrical conductivity of products has been analyzed^43,66^. However, the interphase effects on the percolation threshold and conductivity of graphene system have not been studied.

**Figure 6:**
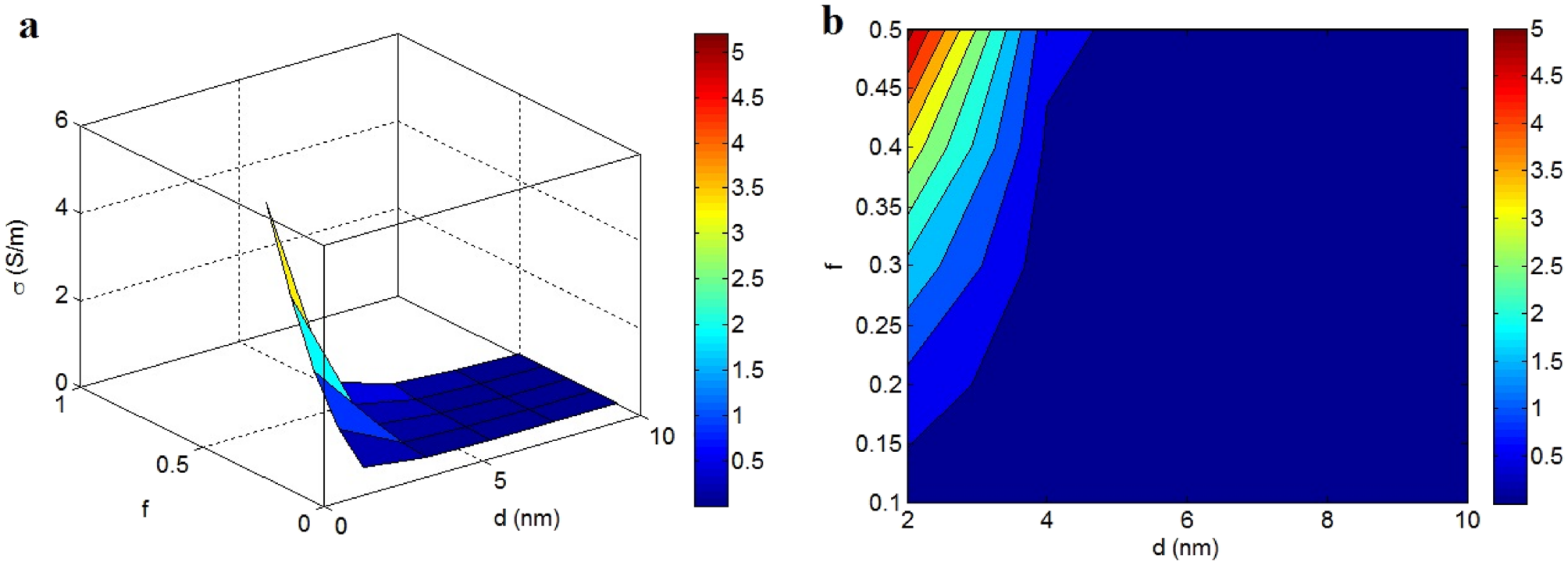
Disparity of conductivity by “d” and “f” applying (**a**) 3D and (**b**) contour configurations.

The conductivity of nanocomposites at various amounts of “d” and “f” and $\phi_{f}$ = 0.01, t = 2 nm, σ_f_ = 10^5^ S/m, t_i_ = 4 nm and K = 2 is depicted in Fig. 6. The high value of “d” and low “f” reduce the conductivity to zero; however, a maximum conductivity of 5 S/m was attained when d = 2 nm and f = 0.5. Consequently, good conductivity was obtained by a short tunneling distance and high percentage of nano-sheets in the nets. However, d > 4.7 nm (at different f) and f < 0.15 (at different d) cause insulating signifying the critical characters of these factors.

The graphene nano-sheets can provide a tunneling effect when the distance between two adjacent nano-sheets does not exceed several nanometers^67,68^. The maximum tunneling distance in polymer CNT nanocomposites has been reported as 10 nm^69^, indicating that a separation distance between nanoparticles below 10 nm can cause the tunneling mechanism and distant graphene nano-sheets to be excluded from the conductivity. Thus, the inverse relation between conductivity and tunneling size is expected, because the distant nano-sheets cannot transport the electrons through the tunneling mechanism.

A high “f” grows the performance and efficiency of nets in the samples, primarily because “f” determines the number of nano-sheets included in the net regions. A large “f” indicates the large and dense nets, while a low “f” exhibits a large number of dispersed nano-sheets in the nanocomposite. Consequently, “f” shows the net size, which controls the conductivity, as recommended by the innovative model.

**Figure 7:**
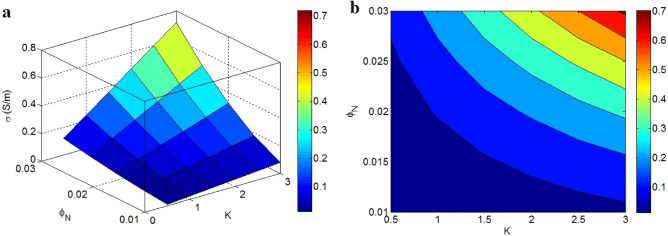
Stimuli of “K” and “$\phi_{N}$” on the conductivity: (**a**) 3D and (**b**) contour schemes.

Figure 7 also illustrates the dependencies of conductivity on “K” and “$\phi_{N}$” (d = 5 nm and σ_f_ = 10^5^ S/m). The uppermost conductivity was obtained as 0.7 S/m at K = 3 and $\phi_{N}$ = 0.03, whereas K < 1 and $\phi_{N}$ < 0.02 resulted in insulation. As a result, both “K” and “$\phi_{N}$” as net parameter and volume fraction of networked nano-sheets confidently manage the conductivity of nanocomposite.

“K” dimensionless parameter represents a function of size and compactness of nets depending on the dispersion and distribution of nanoparticles in the nanocomposite^70^. Undoubtedly, a higher level of “K” shows more scale and concentration of filler nets in the nanocomposite. Since the scale of conductive nets controls the charge transferring in the nanocomposite, it is reasonable to express a direct correlation between the conductivity and “K”. Meanwhile, the volume fraction of conductive nets determines the actual number of nano-sheets, which affects the conductivity of nanocomposite, because some nano-sheets are dispersed in the nanocomposite and do not participate in the nets. Therefore, “$\phi_{N}$” positively governs the conductivity, which verifies the recommended model. According to Fig. 1, several parameters change the “$\phi_{N}$”, which should be optimized to obtain the desired conductivity.

The developed model is applicable for graphene-filled samples with randomly arranged nano-sheets after percolation onset. Moreover, the samples should include the interphase and tunneling parts. The developed model also considers the long and thin nano-sheets dispersed in the nanocomposite. However, the developed model cannot predict the conductivity in samples containing oriented graphene below the percolation onset. Furthermore, the developed model cannot be applied in the absence of interphase and tunneling parts or when the samples contain short and thick nano-sheets.

## Conclusions

An innovative model was established for the conductivity of graphene systems by considering the effects of graphene dimensions, volume fraction of networked sheets, interphase size, tunneling size, and net properties. The recommended model was analyzed by the tested conductivity of samples and factor examination. Both experimented values and factors evaluations ratify the precision of the innovative model. A small percolation threshold and a high graphene conduction produces a desirable conductivity. Moreover, thick nano-sheets meaningfully decrease the conductivity. However, thin and large nano-sheets produce high conductivity. Furthermore, the high levels of filler quantity and interphase deepness significantly enhance the conductivity; however, small and medium levels of these factors result in poor conductivity. An extraordinary conductivity was obtained when the tunneling size was small with a high proportion of nano-sheets in the nets. Furthermore, “K” is representative of net properties and the volume fraction of nets positively affects the conductivity. It is worthwhile mentioning that some factors, such as filler amount, interphase size, tunneling size, and the number of nano-sheets in the nets expressively govern the conductivity. Consequently, these parameters should be managed accordingly to achieve a desirable conductivity in a graphene nanocomposite structure.

## Data Availability

All data generated or analyzed during this study are included in this published article.
